# Plant Fructokinases: Evolutionary, Developmental, and Metabolic Aspects in Sink Tissues

**DOI:** 10.3389/fpls.2018.00339

**Published:** 2018-03-16

**Authors:** Ofer Stein, David Granot

**Affiliations:** Institute of Plant Sciences, Agricultural Research Organization, The Volcani Center, Rishon LeZion, Israel

**Keywords:** fructose, sucrose metabolism, sink strength, xylem development, seed metabolism

## Abstract

Sucrose, a glucose–fructose disaccharide, is the main sugar transported in the phloem of most plants and is the origin of most of the organic matter. Upon arrival in sink tissues, the sucrose must be cleaved by invertase or sucrose synthase. Both sucrose-cleaving enzymes yield free fructose, which must be phosphorylated by either fructokinase (FRK) or hexokinase (HXK). The affinity of FRK to fructose is much higher than that of HXK, making FRKs central for fructose metabolism. An FRK gene family seems to exist in most, if not all plants and usually consists of several cytosolic FRKs and a single plastidic FRK. These genes are expressed mainly in sink tissues such as roots, stems, flowers, fruits, and seeds, with lower levels of expression often seen in leaves. Plant FRK enzymes vary in their biochemical properties such as affinity for fructose, inhibition by their substrate (i.e., fructose), and expression level in different tissues. This review describes recently revealed roles of plant FRKs in plant development, including the combined roles of the plastidic and cytosolic FRKs in vascular tissues and seed development.

## Introduction

Fructokinases (FRKs) are important enzymes that catalyze the key metabolic step of fructose phosphorylation. Unlike mammalian FRKs (also referred to as ketohexokinases), which phosphorylate fructose to form fructose 1-phosphate (F1P), plant FRKs phosphorylate fructose to form fructose 6-phosphate (F6P), similar to bacterial FRKs.

Most of the fructose found in plants originates in carbon assimilated during photosynthesis. In photosynthesis, CO_2_ is fixed in the chloroplasts via the Calvin cycle to yield triose phosphates (triose-P). Triose-P may then be exported to the cytosol, where two triose-P molecules are combined to create one molecule of fructose 1,6-biphosphate (F1,6BP). F1,6BP can be dephosphorylated to form F6P, which is isomerized to yield glucose 6-phosphate (G6P). G6P can be used to form nucleotide sugars such as UDP-glucose (UDP-G). UDP-G and F6P are combined to form sucrose-6-phosphate (sucrose-6P), in a reaction catalyzed by sucrose phosphate synthase, and sucrose-6P is dephosphorylated to yield sucrose – a non-reducing glucose–fructose disaccharide (Dennis and Blakeley, 2000). In many plant species, sucrose is the primary sugar transported from photosynthetic tissues through the phloem to non-photosynthetic tissues (sink tissues), where it serves as a main carbon source for metabolic pathways.

**FIGURE 1:**
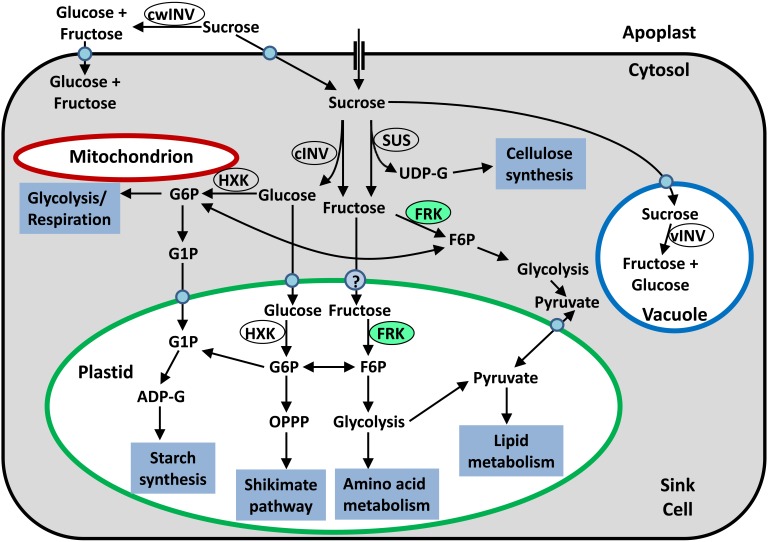
Simplified schematic presentation of sugar metabolism in sink tissue cells. Sucrose may be hydrolyzed in the apoplast by cell-wall invertase (cwINV) to yield glucose and fructose, which can be brought into the cell by a monosaccharide transporter. Alternatively, sucrose can be brought into the sink cell by a sucrose transporter or enter through plasmodesmata. Inside the cell, the sucrose can be stored in the vacuole or hydrolyzed by vacuolar invertase (vINV). In the cytosol, sucrose can be hydrolyzed by cytosolic invertase (cINV) to yield glucose and fructose, or cleaved by SUS to yield fructose and UDP-G. Glucose can be phosphorylated by mitochondria-associated HXK so that it can be used for glycolysis and then respiration, or be brought into the plastids by a plastidic glucose transporter and then phosphorylated by plastidic hexokinase so that it can be fed into plastidic metabolic pathways. Fructose can be phosphorylated by cytosolic FRK (marked in light green) and then used for cytosolic glycolysis or be brought into the plastid by an unknown transporter, phosphorylated by plastidic FRK (marked in light green) and then fed into plastidic metabolic pathways.

The utilization of sucrose for metabolism in sink tissues starts with the cleavage of sucrose into its monosaccharides. Sucrose cleavage is carried out either by invertase (INV) to yield glucose and fructose, or by sucrose synthase (SUS) to yield UDP-G and fructose (Dennis and Blakeley, 2000) (**Figure 1**). While SUS isozymes may be present in the cytosol or mitochondria, or associated with plasma and Golgi membranes (Amor et al., 1995; Carlson and Chourey, 1996; Buckeridge et al., 1999; Dennis and Blakeley, 2000; Winter and Huber, 2000), INV has been localized to the cytosol, cell wall, vacuoles, and plastids (Roitsch and Gonzalez, 2004; Vargas et al., 2008), indicating intercellular compartmentalization and possible differences in the fate of the hexoses released by the cleavage of sucrose.

Before they enter metabolic pathways, the free hexoses glucose and fructose must first be phosphorylated by hexokinase (HXK, EC 2.7.1.1) or FRK (EC 2.7.1.4) to yield G6P and F6P, respectively (**Figure 1**; Dennis and Blakeley, 2000). Glucose can be phosphorylated only by HXK while fructose can be phosphorylated by either HXK or FRK. However, the affinity of HXKs for fructose is generally two orders of magnitude lower than that of FRKs, implying that fructose is primarily phosphorylated by FRKs (Granot et al., 2013). Because fructose accounts for half of the hexose generated by sucrose cleavage in sink tissues, FRKs are considered to be of critical importance for all of the metabolic pathways and formation of organic matter in sink tissues.

## Protein Structure of Plant FRKs

Sugar kinases were initially divided into three major families: the HXKs, the ribokinases, and the galactokinases (Bork et al., 1993). A fourth family called repressor, open-reading framekinase (ROK) was found to include a number of sugar kinases, including FRKs, primarily in bacteria (Thompson et al., 1991; Zembrzuski et al., 1992; Sato et al., 1993; Titgemeyer et al., 1994; Nocek et al., 2011). To date, all plant FRKs have been assigned to the phosphofructokinase type B (pfkB) subfamily, a large group within the ribokinase family, based on their sequence similarity with the first pfkB gene, *Pfk-2*, the minor pfk from *Escherichia coli* (Morrissey and Fraenkel, 1972). The pfkB group consists mainly of carbohydrate, phosphocarbohydrate, and pyrimidine kinases (Gilkerson et al., 2012). Some of the pfkB proteins found in plants do not possess kinase activity, but rather play a role in redox signaling and gene expression, mainly in chloroplasts. The Arabidopsis FRK-like enzymes 1 and 2 (FLN1 and FLN2) are examples of this (Arsova et al., 2010). These proteins share substantial sequence similarity with known FRKs, yet they do not possess any FRK activity. FLN1 and FLN2 were found to interact with thioredoxin z to control plastidic gene expression (Arsova et al., 2010).

**FIGURE 2:**
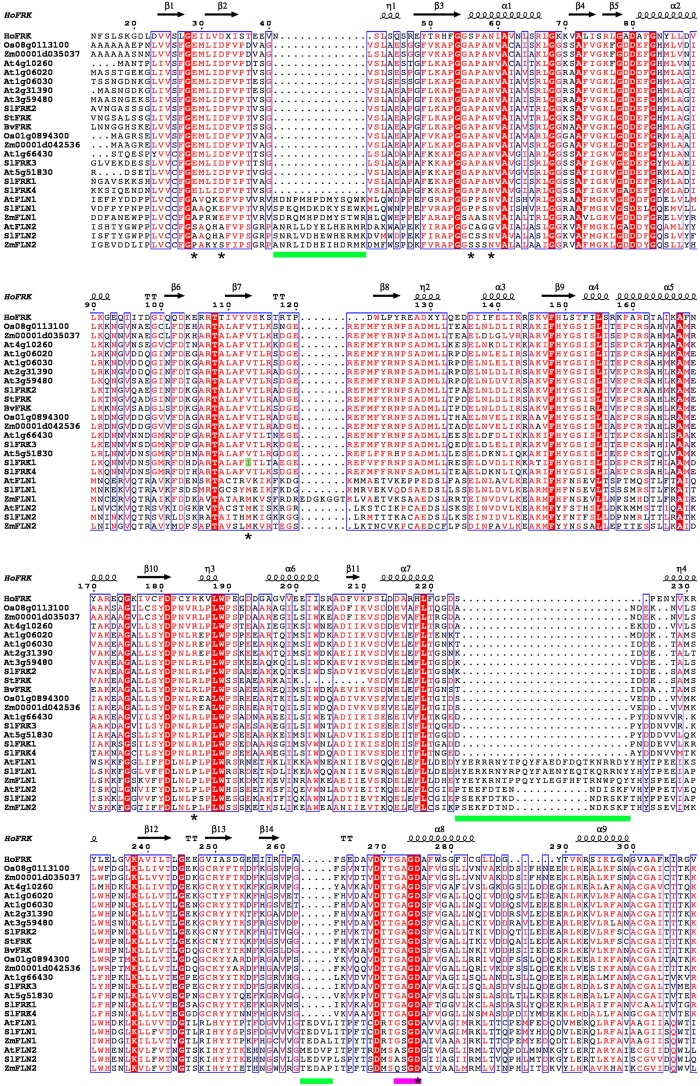
Sequence alignment of plant FRKs and FLNs. Sequence alignment was carried out using ClustalW and the figure was created using ESPript 3.0 (Robert and Gouet, 2014). The structure of *H. orenii* FRK (PDB – 3HJ6) was used as a reference structure. N-terminal and C-terminal regions were excluded from the graphic presentation. A red background indicates amino acid sequence identity and red letters indicate sequence similarity. Green bars show the locations of insertions in FLNs. A pink bar is used to indicate the G/AXGD motif. Asterisks indicate suggested amino acids with importance for substrate binding (Chua et al., 2010). Accession IDs of the proteins used for the sequence alignment: SlFRK1 (AAB57733), SlFRK2 (AAB57734), SlFRK3 (NP_001234396), SlFRK4 (AAM44084), At1g06020 (AAF80125), At1g06030 (AAF80126), At1g66430 (AAG51160), At2g31390 (AAM14251), At3g59480 (CAB75445), At4g10260 (CAB78149), At5g51830 (AAL34211), Os01g0894300(AAL26574), Os08g0113100 (AAL26573), Zm00001d035037 (AAP42806), Zm00001d042536 (AAP42805), BvFRK (AAA80675), StFRK (CAA78283), AtFLN1 (AEE79187), AtFLN2 (NP_177080), SlFLN1 (XP_004246362), SlFLN2 (XP_004239035), ZmFLN1 (ONM36391),and ZmFLN2 (ONM06924).

To better understand what differentiates plant FRKs from FLNs, we compared the amino acid sequences of 17 confirmed active FRKs and the Arabidopsis FLN1 and FLN2 and their homologs from tomato (*Sl*) and maize (*Zm*; **Figure 2**). A sequence identity matrix (**Supplementary File S1**) shows that the minimal shared identity between any pair of the 17 active FRKs is 49.3%, whereas the maximal shared identity between one of the examined FLNs and any of the active FRKs is 26.4%, indicating quite a significant difference between the FRKs active in plants and the examined FLNs.

The structure of a ribokinase from *E. coli* (1RKD), determined by X-ray crystallography (Sigrell et al., 1998), was used as a model for the structure of pfkB proteins. pfkB proteins contain two main domains, a large active site composed of a β-sheet positioned between two α-helices and a smaller, peripheral β-sheet known as the lid domain. Many pfkB carbohydrate kinases act as homodimers and the interaction between monomers occurs mainly across the lid domain (Riggs and Callis, 2017). There are two main motifs shared by all pfkB proteins. The first is an N-terminal pair of glycines that is located at a hinge between the two domains and the second is a G/AXGD involved in catalysis. Interestingly, in all 17 FRKs active in plants, the second motif is GAGD, whereas in FLN1 it is GSGD and in FLN2 it is A/QSGD (**Figure 2**). Another significant difference between plant FRKs and FLNs is their length. FRKs that are active in plants are between 319 amino acids (StFRK) and 389 amino acids long (SlFRK3, which includes a chloroplast transit peptide), whereas FLN1 is about 500 amino acids long and FLN2 is >600 amino acids long. The difference in length between plant FRKs and these FLNs is due to the FLNs longer N-terminal sequence (with unknown function) and at least three additional insertions (**Figure 2**). A protein-structure model has predicted that although these proteins share characteristics with active FRKs, the additional N-terminal sequence and insertions may create loops that might interfere with fructose binding (Riggs and Callis, 2017).

The crystal structure of a FRK from the anaerobic, thermohalophilic bacterium *Halothermothrix orenii* was used to develop a model for the activity of plant FRKs, by comparing that bacterial protein with the homologous enzyme from tomato (SlFRK1). Although other bacterial FRK structures have been resolved previously, the *H. orenii* FRK shares more sequence identity with plant FRKs, allowing better structure predictions. Based on this model, the plant FRK residues that might be important for the fructose-binding site are Glu33, Asp37, Ala60, Asn63, Ile117, Arg192, and Asp285 (Chua et al., 2010). These seven amino acids are 100% conserved in all 17 active FRKs (marked with an asterisk in **Figure 2**), with the exception of Ile117, which is unique to SlFRK1. In contrast, all of the other active FRKs contain valine in this position (letter highlighted in green, **Figure 2**). These seven amino acids are not conserved in the six FLNs examined, with the exception of the second aspartate in the G/AXGD motif (**Figure 2**). The substitution of valine for isoleucine 117 in SlFRK1 might explain its relatively low affinity for fructose (1.3 mM compared to <0.1 mM for the other tomato FRKs). In any case, bacterial FRKs’ homology to plant FRKs is still relatively low and X-ray crystallography of an active plant FRK together with fructose and site-directed mutagenesis can be very useful tools for learning about the structure of plant FRKs.

## Plant FRK Gene Families and Phylogeny

Due to partial sequence similarity with other pfkB proteins, it has been difficult for protein-identification algorithms to define which plant genes are genuine FRKs based only on sequence data and enzymatic assays of FRK activity may be required. The FRK gene families have been studied comprehensively in tomato and Arabidopsis. In tomato, four genes were cloned and designated *SlFRK1–4*. The FRK activity of the four *SlFRK* genes was confirmed by expression in a yeast triple-mutant (DFY-632), which lacks fructose and glucose phosphorylation activity and cannot utilize glucose or fructose. The tomato FRK genes complemented the ability of that yeast mutant to grow on media containing fructose, but not on media containing glucose, indicating genuine FRK activity (Kanayama et al., 1997, 1998; German et al., 2002, 2004). The SlFRK isozymes were characterized *in vitro* through expression in yeast and the analysis of protein extracts from the transformed yeasts, further confirming their catalytic FRK activity (Petreikov et al., 2001; German et al., 2002, 2004). Interestingly, sequencing of the tomato genome revealed a probable fifth FRK gene (FRK5, Solyc11g042850). However, based on RNA-seq data, the expression of this gene is very limited and, therefore, it was probably not detected prior to the genome sequencing (Sato et al., 2012; Koenig et al., 2013).

In Arabidopsis, seven FRK genes (At5g51830, At2g31390, At1g66430, At4g10260, At1g06020, At1g06030, and At3g59480) were identified and named *AtFRK1–7*, respectively (Riggs et al., 2017). The identities of the Arabidopsis FRKs were verified following protein expression in *E. coli*, purification and comprehensive characterization of their biochemical and enzymatic properties (Riggs et al., 2017). Previous studies used somewhat different numbering for some of the Arabidopsis FRKs (Pego and Smeekens, 2000; Stein et al., 2017a), but The Arabidopsis Information Resource (TAIR) recently decided to adopt the annotations created by Riggs et al. (2017), which are more compatible with the numbering used in earlier publications (Pego and Smeekens, 2000; Arsova et al., 2010). In cassava (*Manihot esculenta*), the FRK gene family was identified using bioinformatic tools and seven individual FRK genes were identified. Two of those genes (*MeFRK3* and *MeFRK4*) were confirmed to be FRKs by complementation of the yeast mutant’s ability to grow on media containing fructose, but not on media containing glucose (Yao et al., 2017).

An attempt to characterize the FRK gene family in sugarcane (*Saccharum spontaneum*) using only bioinformatic tools appears to have been less successful. Although it suggests the presence of seven FRK genes in sugarcane (*SsFRK1–7*), a phylogenetic tree based on protein sequences that includes the Arabidopsis FRKs shows that only SsFRK1–2 are found in the same clades with the seven confirmed Arabidopsis FRKs (Chen et al., 2017). SsFRK3 and SsFRK5 are found in the same clades as At1g69200 (FLN1) and At3g54090 (FLN2; Chen et al., 2017) and, therefore, are probably FRK like and less likely possess FRK activity. In contrast, SsFRK4, SsFRK6, and SsFRK7 are found on even more distant branches together with other Arabidopsis pfkB proteins that are not FRKs (At1g06730, At1g49350, and At4g28706, respectively; Chen et al., 2017). This may indicate that only SsFRK1 and SsFRK2 are indeed genuine FRKs, in agreement with the fact that only two FRK enzymes have been characterized in sugarcane (Hoepfner and Botha, 2003, 2004). A similar analysis performed with tea plants (*Camellia sinensis*) found seven FRK genes (*CsFRK1–7*; Li et al., 2017). However, a phylogenetic tree constructed using those FRK amino acid sequences revealed that CsFRK5–7 are in the same clade as Arabidopsis FLN1 and FLN2 (Li et al., 2017) and, therefore, are less likely to be genuine FRKs. Yet, CsFRK1–4 are found in the same clades as all of the other active Arabidopsis FRKs (Li et al., 2017), suggesting that tea has four genuine FRKs.

**FIGURE 3:**
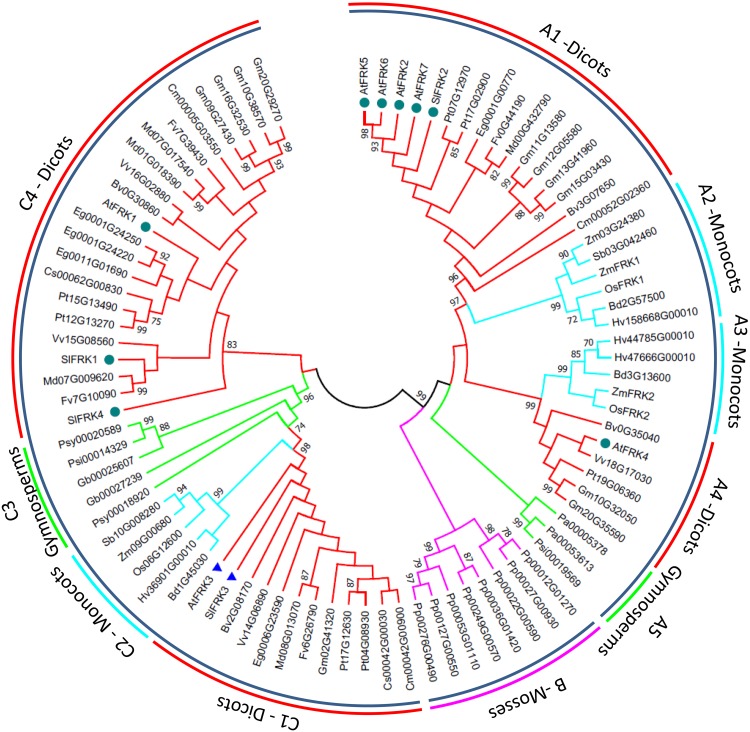
Phylogenetic tree of FRK genes from land plants. Amino acid sequences were retrieved using the Plaza 3.0 tool for gene family analysis (Van Bel et al., 2017). Partial sequences and sequences identified as FRK-like (FLNs) based on their length and insertions were excluded, leaving a total of 88 sequences. Confirmed FRK genes were renamed based on previous annotations. Sequences were aligned using ClustalW with default options and analyzed in MEGA 7.0 (Kumar et al., 2016). The tree was created using the maximum-likelihood method based on the JTT matrix-based model (Jones et al., 1992). Bootstrap values >70% are denoted at the nodes. The pink branches belong to the mosses. Green branches belong to in the gymnosperms. Turquoise branches belong to the monocots and red branches belong to the dicots. Dark green circles indicate FRKs that were confirmed to be cytosolic and blue triangles indicate FRKs that were confirmed to be plastidic.

In order to get a better picture of how the FRK gene family looks in other plant species, we used the PLAZA3.0 program^1^ to retrieve FRK protein sequences from dicots, monocots, gymnosperms, and the moss *Pp*. Potential FRK sequences were manually selected and partial sequences were removed, as well as sequences that were identified as FRK-like based on their length and insertions, leaving 88 sequences, which we used to create a broad phylogenetic tree for plant FRKs (**Figure 3**). This phylogenetic tree shows that plant FRKs can be divided into three distinct groups: A, B, and C. All *Pp* FRKs are in one clade (B) that is composed of two sub-groups that are separated from other plants (pink branch in **Figure 3**), as was previously shown (Riggs et al., 2017). Interestingly, type A FRKs are found among one group of gymnosperms (A5) and two groups of angiosperms (A1, A2 and A3, A4), and each angiosperm group is divided into one monocot and one dicot subgroup (A1-monocot, A2-dicot and A3-monocot, A4-dicot). This suggests that FRK gene duplication and speciation events occurred in an angiosperm ancestor.

Five of the seven Arabidopsis FRKs are type A FRKs, with four proteins (AtFRK2, AtFRK5-7) in the A1 subgroup and one (AtFRK4) is in the A4 subgroup (**Figure 3**). The type C FRKs can be divided into four groups, in which C1, C2, and C3 (dicots, monocots, and gymnosperms, respectively) are bunched together, and the C4 group, which is unique to dicots (**Figure 3**). The C1 group contains the only two confirmed plastidic FRKs, the Arabidopsis FRK3 (At1g66430) and the tomato SlFRK3 (marked with blue rectangles, **Figure 3**), suggesting that C1 is probably in the plastidic clade and raising the possibility that the C2 and C3 clades may also represent plastidic FRKs in monocots and gymnosperms, respectively. The other confirmed cytosolic FRKs (marked with dark green circles in **Figure 3**) are present in A1, A4, and C4, suggesting that type A FRKs and type C4 FRKs are probably cytosolic.

## Enzymatic Activity of Plant FRKs

Plant FRKs usually act as homodimers with a monomer mass of about 34–37 kDa (see review by Pego and Smeekens, 2000). FRKs generally utilize ATP as the phosphate donor for the phosphorylation of fructose, due to their high affinities for ATP, but may use other nucleotides such as GTP and UTP in the absence of ATP (Pego and Smeekens, 2000). Some FRKs have higher levels of activity with GTP (Doehlert, 1990; Gardner et al., 1992; Hoepfner and Botha, 2004) or CTP (Doehlert, 1990).

Plant FRKs usually have pH optima of about 8.0 (Pego and Smeekens, 2000). Magnesium ions (Mg^2+^) are required for plant FRK activity (Turner et al., 1977; Renz and Stitt, 1993; Schaffer and Petreikov, 1997a; Gonzali et al., 2001; Karni and Aloni, 2002), but those ions can sometimes be partially replaced by manganese ions (Mn^2+^; Turner et al., 1977; Copeland and Morell, 1985). Potassium ions (K^+^) have been reported to stimulate plant FRK activity (Turner et al., 1977; Copeland et al., 1978; Copeland and Morell, 1985; Baysdorfer et al., 1989; Gardner et al., 1992).

Plant FRKs have a wide range of affinities for fructose ranging from *K*_m_ = 0.006 mM for FRKIb of barley (*Hv*; Baysdorfer et al., 1989) to *K*_m_ = 3.3 mM for OsFKI of rice (*Os*; Jiang et al., 2003, and the review by Pego and Smeekens, 2000). Only a few plant FRKs have *K*_m_ values >1 mM and the affinity of most plant FRKs for fructose is about two orders of magnitude greater than the affinity of plant HXKs for fructose (Granot et al., 2013). In Arabidopsis, all seven FRKs have been characterized and their affinities for fructose range from 0.012 to 0.48 mM. Their affinities for ATP range from 0.052 to 0.28 mM and seem to be negatively correlated with their respective affinities for fructose. AtFRK5 and AtFRK7 were found to have low turnover rates (kcat of 4.8 and 1.5, respectively), whereas the other Arabidopsis FRKs have turnover rates of 10.3–14.3 kcat (Riggs et al., 2017).

Another important aspect of plant FRK activity is its inhibition by its own substrate, fructose. Many plant FRKs, including plastidic FRKs, have been reported to be inhibited by fructose concentrations above 1–2 mM, with *K*_i_ values of 1–6 mM (Pego and Smeekens, 2000). It appears that FRKs with higher affinities for fructose are usually more susceptible to substrate inhibition than FRKs with lower affinities (Pego and Smeekens, 2000). Because fructose also inhibits the cleavage of sucrose by SUS in a product-inhibition manner, it has been suggested as a mechanism for the regulation of carbohydrate flux into starch synthesis in young tomato fruits (Schaffer and Petreikov, 1997b) and during vascular development (German et al., 2003; Damari-Weissler et al., 2009). Fructose substrate inhibition might also play a role in directing carbon to different metabolic pathways. Because some FRKs are inhibited by high concentrations of fructose and some are not and because FRKs may also differ in their intracellular localization (cytosol or plastids), it is possible that the accumulation of fructose in the cytosol may restrict the allocation of fructose to cytosolic glycolysis, increasing the availability of fructose for plastidic metabolic pathways such as glycolysis in the organelle, the oxidative pentose phosphate pathway, starch synthesis, and the shikimate pathway (**Figure 1**; Stein et al., 2016, 2017a).

## Subcellular Localization of Plant FRKs

The subcellular localization of FRKs has not been thoroughly investigated and data regarding the compartmentalization of the enzyme are scarce. The results of fractionation by differential centrifugation and a sucrose density gradient indicate that, in pea (*Pisum sativum*) stems, FRK activity is located exclusively in the cytosol (Tanner et al., 1983), whereas a biochemical study of plastids isolated from spinach suggested that this enzyme is found in plastids (Schnarrenberger, 1990).

The first work to address the plant subcellular localization of the FRK gene family was done with the four tomato FRKs, *SlFRK1-4*. Using green fluorescent protein (GFP) fused to *SlFRK1-4* proteins expressed in tobacco protoplasts, it was found that while SlFRK1, SlFRK2 and SlFRK4 are cytosolic enzymes, SlFRK3 is located in the chloroplast stroma (Damari-Weissler et al., 2006). A GFP fusion of SlFRK3 missing the first 30 amino acids was found in the cytosol, indicating that SlFRK3 contains a transit peptide that directs it to the chloroplast (Damari-Weissler et al., 2006). In another study aimed at characterizing the seven FRK genes in Arabidopsis, researchers observed the transient expression of AtFRKs fused to yellow fluorescent protein (YFP) in tobacco (*Nicotiana benthamiana*) leaves. That study showed that *AtFRK3* (At1g66430) is a plastidic FRK, whereas the other six FRKs are located in the cytosol (Riggs et al., 2017). These observations involving tomato and Arabidopsis genes suggest that other plant species may also have a single plastidic FRK and several cytosolic FRKs.

## Expression Patterns of Plant FRKs

The patterns of FRK gene expression in different organs have been examined in only a few plant species. Expression analysis of FRK genes in cassava, carried out using qPCR, showed that *MeFRK1–4* are expressed in leaves, stems, tubers, flowers, and fruits (although *MeFRK2* expression was relatively low), while *MeFRK5* is specific to flowers and *MeFRK6* is specific to leaves and expressed at very low levels (Yao et al., 2017). *MeFRK3* and *MeFRK4* are highly expressed during early cassava tuber development and their expression is correlated with high levels of FRK activity (Yao et al., 2017). Expression analysis, by qPCR, of a FRK gene in loquat (*Eriobotrya japonica*) revealed expression in leaves, stem, flowers, and fruits (Qin et al., 2014). In corn (*Zm*), *ZmFRK1* and *ZmFRK2* expression were detected, by Northern blot analysis, mainly in roots, stems, and developing seeds and was hardly detected in leaves, suggesting the importance of these genes in the metabolism of sink tissues (Zhang et al., 2003). In rice, Northern blotting revealed *OsFKI* expression in roots, endosperm, and leaf tissues, whereas *OsFKII* was detected in roots and endosperm and, at low levels, in leaves (Jiang et al., 2003).

Expression analysis of all four tomato *SlFRKs* by qPCR revealed that *SlFRK1–3* are expressed in different levels in all organs, whereas *SlFRK4* is expressed only in anthers and stamens (German et al., 2004). Better resolution of tomato FRK expression was achieved at the tissue level using transgenic plants expressing the FRKs promoters fused to the GUS reporter gene. *SlFRK4* promoter GUS staining revealed expression at late stages of pollen development and pollen germination (David-Schwartz et al., 2013), whereas the expression of *SlFRK1–3* promoters was observed primarily in secondary vascular tissues. The *SlFRK2* promoter is expressed throughout the secondary xylem, whereas the *SlFRK1* promoter is expressed mainly in mature xylem fibers (Stein et al., 2017b). *SlFRK3*, the tomato plastidic FRK, is expressed in the cambium and in developing xylem fibers (Stein et al., 2016). In addition, all three promoters are expressed in phloem companion cells (Stein et al., 2017b). Although all three promoters are also expressed in leaves, GUS staining revealed expression only in the leaf veins and vascular tissue (Stein et al., 2016, 2017b). These expression patterns further suggest a more important role for FRKs in sink tissues, compared to photosynthetic tissues.

Fructokinase gene expression is also affected by different external and internal factors. Some FRK genes show increased expression in response to sugars. In tomato cotyledons, both *SlFRK1* and *SlFRK2* show elevated mRNA levels in response to treatment with glucose, fructose, or sucrose (Kanayama et al., 1998). Real-time PCR analysis of loquat FRK also revealed a response to fructose or glucose (Qin et al., 2014). In rice, *OsFK2* expression is induced by anoxia, while *OsFK1* expression is reduced by anoxia (Guglielminetti et al., 2006). Under anoxic conditions, similar patterns were also detected in the protein levels of OsFK2 and OsFK1 in the coleoptile and embryo, but not in roots (Guglielminetti and Volterrani, 2014). In roots, levels of both OsFK2 and OsFK1 proteins were drastically reduced under anoxic conditions (Guglielminetti and Volterrani, 2014). Other plant FRKs also respond to other types of abiotic stress, including salt stress, drought, and wounding (Klotz et al., 2006; Fulda et al., 2011; Zorb et al., 2011; Li et al., 2017). The different patterns of expression, subcellular localization, and substrate inhibition observed among plant FRKs suggest that these enzymes may play important roles in directing carbohydrate metabolism toward distinct metabolic pathways and in regulating the amount of carbohydrate metabolized under various environmental conditions, especially in sink tissues. The increase in the availability of expression data (primarily RNA-seq and microarray data) from many plant species in recent years will allow for more accurate speculations regarding the roles of specific FRKs in plant development.

## Roles of Plant FRKs in Development

Due to the strong correlation between SUS and FRK enzyme activity and starch accumulation in young tomato fruits, it has been suggested that FRK might play a role in supplying carbon for starch accumulation in young tomato fruits (Schaffer and Petreikov, 1997b). However, an analysis of transgenic tomato plants with antisense suppression of FRK did not reveal any effects on starch synthesis in young fruits (Dai et al., 2002; Odanaka et al., 2002). In potato (*Solanum tuberosum*), antisense suppression of *StFK1* did not significantly alter leaf carbon metabolic enzyme activity or metabolite levels (Davies et al., 2005). However, altered FRK activity in developing potato tubers was associated with increased sucrose levels. When metabolic flux was estimated using radiolabeled fructose, it became apparent that FRK activity affects the rate of redistribution of radiolabeled carbon to sucrose, suggesting that the FRK enzyme might maintain a balance between sucrose degradation and synthesis and might work, together with SUS, to maintain sink strength (Davies et al., 2005).

Some of the more recent research has demonstrated the importance of plant FRKs for the development of vascular tissues. Suppression of tomato *SlFRK2* reduced the area of xylem vessels, forming deformed small secondary xylem vessels with thin cell walls, which reduced hydraulic conductivity in stems, roots, and leaves (Damari-Weissler et al., 2009; German et al., 2003). It also resulted in narrow, short phloem sieve elements, which reduced sugar transport (Damari-Weissler et al., 2009). Specific RNAi suppression of the tomato plastidic FRK, *SlFRK3*, had no visible growth effects, but did cause a slight reduction in the hydraulic conductivity of stems and roots (Stein et al., 2016). However, when *SlFRK3* was co-suppressed together with *SlFRK2*, plant growth was severely inhibited, mature leaves wilted, and fruit-setting and seed-setting were compromised (Stein et al., 2016). Anatomical analysis revealed that co-suppression of *SlFRK3* and *SlFRK2* resulted in smaller secondary xylem fibers with thin cell walls that occasionally collapsed, indicating the importance of both FRKs for xylem–fiber development (Stein et al., 2016). A similar pattern was detected using transgenic plants with antisense suppression of *SlFRK1*. Suppression of *SlFRK1* alone reduced hydraulic conductance in the stem, but not in roots and did not have any visible effects. Combined suppression of *SlFRK1* and *SlFRK2* resulted in severe growth inhibition very similar to that caused by the combined suppression of *SlFRK3* and *SlFRK2* (Stein et al., 2017b). Anatomical analysis revealed a smaller xylem area with reduced numbers of xylem vessels and small phloem fibers with thin cell walls, indicating the combined importance of FRK1 and FRK2 for phloem–fiber development (Stein et al., 2017b). The observed effects of the three tomato FRKs on the xylem and phloem cell-wall width suggest the importance of FRKs for cell-wall metabolism. This suggestion is also supported by results obtained in aspen wood (*Populus tremula* × *P. tremuloides*), where reduced FRK activity led to narrower xylem fibers with reduced cellulose content, indicating that FRK is important for carbon partitioning to cellulose (Roach et al., 2012).

In Arabidopsis, quadruple- and penta-mutants showed similar phenotypes to those induced by the co-suppression of tomato *SlFRK2* and *SlFRK3*, exhibiting reduced cambium activity, reduced xylem area, narrow vessels with thin cell walls, and dark necrotic lesions around the cambium (Stein et al., 2017a). This indicates that Arabidopsis and tomato FRKs play similar roles in vascular development.

Interestingly, an Arabidopsis double-mutant possessing a mutation in the plastidic FRK (At1g66430) and a cytosolic FRK (At5g51830) had a seed-specific phenotype. The double-mutant seeds were wrinkled, with an abnormal seed surface, and weighed less than WT seeds (Stein et al., 2017a). The abnormal seeds also exhibited growth arrest after germination that could be rescued by the addition of glucose or sucrose to the growth media, suggesting that those seeds might be lacking some of the storage reserves required for seedling establishment (Stein et al., 2017a). Transmission electron microscopy revealed that the double-mutant embryo cells had smaller oil bodies and seed fatty acid analysis revealed a 20–50% decrease in the major fatty acids in those seeds, indicating reduced oil accumulation (Stein et al., 2017a). This phenotype of the double-mutant of plastidic and cytosolic FRKs and the absence of any unique phenotype for any of the single mutant lines indicate that fructose phosphorylation by either FRK1 or FRK3 (FRK7 or FRK6, respectively, in Stein et al., 2017a) is necessary for seed oil accumulation. This also indicates that fructose may enter plastids efficiently, probably by carrier-mediated transport, but a plastidic fructose transporter has yet to be identified (Schafer and Heber, 1977; Stein et al., 2017a). The importance of FRKs for seed development was also noted in tomato. *In situ* hybridization of *SlFRK1* and *SlFRK2* during early fruit development revealed that while FRK1 mRNA is found in most of the seed and placental cells, FRK2 mRNA is located primarily in the endosperm (Kanayama et al., 1998). In addition, suppression of *SlFRK2* in tomato reduced the number of seeds per fruit, indicating the importance of fructose metabolism for early seed development (Odanaka et al., 2002). Combined suppression of tomato *SlFRK2* with *SlFRK1* or with *SlFRK3* yielded very stunted plants that did not set fruit at all (Stein et al., 2016, 2017b), making it difficult to study the effect of combined suppression of FRKs on seed development, as was done in Arabidopsis.

Taken together, these results indicate the importance of plant FRKs for carbon metabolism, primarily in sink tissues and, more specifically, for vascular and seed development.

## Manipulation of FRK Activity to Improve Important Agricultural Traits

Since FRKs are key players in carbon metabolism, they could potentially be used to target important agricultural traits such as yield, fruit sugar content and composition, and cell-wall composition. Only a handful of studies have examined the effect of FRK overexpression in plants. Overexpression of potato FRK (*StFK*) in potato and tomato plants did not reveal any significant beneficial effects (German et al., 2003; Davies et al., 2005). On the other hand, overexpression of tomato *SlFRK1* in cotton (*Gossypium hirsutum*) increased the number of cotton bolls per plant, the number of seeds per boll, and fiber mass per plant, without affecting fiber length or fiber strength (Mukherjee et al., 2015). It has been suggested that these phenotypes result from the increased leaf area of the *SlFRK1*-overexpressing plants at the time of flowering, which allows for greater carbon assimilation per plant (Mukherjee et al., 2015). Further research is required to demonstrate any positive effects of FRK overexpression, possibly in a tissue-specific manner and perhaps together with SUS, as it has been suggested that FRKs and SUS may work in concert to regulate sucrose metabolism (Davies et al., 2005).

## Regulatory Roles of Plant FRKs

Sugars such as sucrose, trehalose 6-phosphate, glucose, and fructose are not only required for metabolism, but may also act as signaling molecules controlling plant growth and development (Sheen et al., 1999; Rolland et al., 2006; Ruan, 2014; Figueroa and Lunn, 2016). It was suggested that plant FRKs might be important for fructose-sensing and signal transduction (Pego and Smeekens, 2000), perhaps in a manner similar to HXK in the context of glucose-sensing (Jang et al., 1997). However, although growth-inhibited Arabidopsis seedlings on artificial media supplemented with 6% glucose or fructose exhibited similar phenotypes, fructose-sensing was not shown to be related to FRK or HXK. Instead, fructose-sensing was found to be mediated by fructose 1,6-bisphosphatase and the transcription factor FSQ6/ANAC089 (Cho and Yoo, 2011; Li et al., 2011).

However, recent work has revealed a possible regulatory role for FRKs in determining the flowering time of Arabidopsis. Plants with mutated *AtFRK3* (the plastidic FRK) and *AtFRK1* exhibited slightly delayed flowering under short-day conditions (Jin et al., 2017). TWIN SISTER OF FT (TSF) is a homolog of FT (Flowering Locus T) and is thought to play an important role in the regulation of flowering under short-day conditions. Yeast two-hybrid, pull-down, and bimolecular fluorescence complementation (BiFC) assays revealed a protein–protein interaction between AtFRK3 and TSF (Jin et al., 2017). TSF was localized to the nucleus using TSF–GFP fusion in protoplasts, while AtFRK3 was found mainly in chloroplasts, but also in the nucleus and BiFC revealed an interaction between the two proteins in the nucleus (Jin et al., 2017). Although the mechanism is not entirely clear, it has been suggested that the interaction of AtFRK3 with TSF may inhibit its FRK activity (Jin et al., 2017).

Similar to the flowering-time effects observed among *AtFRK3* and *AtFRK1* knock-outs, suppression of tomato SlFRK1 (but not SlFRK2) also delayed flowering time (Odanaka et al., 2002). Interestingly, *AtFRK1* and *SlFRK1* are in the same dicot-only branch of the phylogenetic tree (**Figure 3**), suggesting that other dicot FRKs might also be involved in the regulation of flowering time.

## Plant FRK Functions Are Partially Redundant

With the exception of tomato *SlFRK2*, whose suppression causes severe growth inhibition (Odanaka et al., 2002; German et al., 2003), no transgenic tomato line with reduced expression of a single FRK or Arabidopsis single-gene knock-out mutant showed substantially altered growth, suggesting significant redundancy among the FRKs (Stein et al., 2016, 2017a,b; Riggs et al., 2017).

Progeny of the tomato FRK2-antisense line crossed with either the FRK1 or FRK3 lines mainly had altered xylem–fiber and phloem–fiber development, respectively. This indicates some level of redundancy of FRK2 and FRK3 in xylem–fiber development, and of FRK2 and FRK1 in phloem–fiber development (Stein et al., 2016, 2017b). Similar redundancy was observed in Arabidopsis, when only double-mutants exhibited the seed-development phenotype, while quadruple- and penta-mutants also exhibited vascular tissue-related alterations in phenotype (Stein et al., 2017a). The redundancy observed in the function of tomato and Arabidopsis FRKs also raises the possibility that some fructose may be phosphorylated by HXK. Although FRKs are high-affinity fructose-specific phosphorylating enzymes, HXKs are also capable of fructose phosphorylation though at much lower affinity than FRKs. It is possible, therefore, that in the absence of FRK, some fructose phosphorylation is carried out by HXK, which may also explain some of the redundancy in FRK functions in tomato and Arabidopsis. We speculate that phenotypes detected in plants with multiple altered FRKs involve cells of tissues that are strong sinks, such as developing xylem vessels and fibers, in which rapid rates of fructose phosphorylation and metabolism are needed to support rapid growth and, possibly, the formation of thick, rigid cell walls.

## Summary and Avenues for Future Work

Sugars are the main input for all metabolic pathways and hexose (glucose and fructose) phosphorylation is essential for hexose metabolism. Only two group of hexose-phosphorylating enzymes exist in plants, HXKs and FRKs. While HXKs have been extensively studied, the study of FRKs has lagged slightly behind. However, recent studies have shown that FRKs are indispensable for carbon metabolism in sink tissues such as the vasculature and seeds. Many plant species have a single HXK and a single FRK in their plastids. The importance of HXK for plastidic sugar metabolism is not yet clear, but plastidic FRKs appear to be involved in fatty acid metabolism. The distinct roles of cytoplasmic HXKs and FRKs might be more evident considering their different intracellular localization. In most plant species, cytoplasmic HXKs are associated with the mitochondria while FRKs are located in the cytosol. This raises the possibility that phosphorylated glucose is routed primarily to the mitochondria while fructose phosphorylated by FRK is fed into cytoplasmic pathways, some of which may be aimed at cell-wall formation, with specific functions in xylem vessels, fibers, and overall vasculature development. Specific FRK isozymes are inhibited by their own substrate (fructose), a phenomenon that limits the amount of fructose (and total sugar) that can be fed into a given pathway. Accordingly, increasing FRK activity in a specific sink tissue, together with SUS, might increase sink strength and improve valuable agricultural traits like seed oil content and fatty acid composition, enhance cambium activity, and/or increase wood production.

Future studies may be guided by the following three questions: (1) which protein is involved in the transport of fructose between the cytosol and plastids? (2) What is the importance of fructose phosphorylation versus glucose phosphorylation, and of cytoplasmic versus plastid fructose phosphorylation, and how is the compartmentalization of the substrates achieved? (3) Can FRK activity in sink tissues be manipulated to alter traits such as yield and sugar content? Although no sugar-sensing function has yet been attributed to FRKs, the different forms of this enzyme appear to be indispensable for plant development.

## Author Contributions

OS wrote this manuscript with revisions and editorial advice from DG.

## Conflict of Interest Statement

The authors declare that the research was conducted in the absence of any commercial or financial relationships that could be construed as a potential conflict of interest.

## Footnotes

1 https://bioinformatics.psb.ugent.be/plaza/

## Supplementary Material

**FILE S1 |** Sequence identity matrix among the amino acid sequences of active FRKs from six plant species, and of FLN1 and FLN2 from three plant species. The protein sequences that were used to create the identity matrix using BioEdit software are similar to those of **Figure 2**. Values marked in green are above 49% identity and values marked in red are below 30% identity.

## Abbreviations

At: Arabidopsis thaliana
Sl: Solanum lycopersicum
Pt: Populus trichocarpa
Gm: Glycine max
Md: Malus domestica
Cm: Cucumis melo
Fv: Fragaria vesca
Vv: Vitis vinifera
Bv: Beta vulgaris
Eg: Eucalyptus grandis
Cs: Citrus sinensis
Pp: Physcomitrella patens
Os: Oryza sativa
Hv: Hordeum vulgare
Zm: Zea mays
Bd: Brachipodium distachion
Sb: Sorghum bicolor
Pa: Picea abies
Psi: Pinus sitchensis
Psy: Pinus sylvestris
Gb: Ginkgo biloba
